# *threo*-Diethyl 2-ethyl-2-hy­droxy-3-(4-methyl­benzene­sulfonamido)­succinate

**DOI:** 10.1107/S1600536811029527

**Published:** 2011-07-30

**Authors:** Sofiane Mekki, Valérie Rolland, Arie van Lee, Marc Rolland

**Affiliations:** a Laboratoire de Synthèse Organique Appliquée , Université d’Oran Es-Sénia, Département de Chimie, BP 1524, El Ménouer, Oran 31000, Algeria; b Institut des Biomolécules Max Mousseron, UMR 5247, CNRS, UM2, UM1, Place E. Bataillon, 34095 Montpellier, Cedex 5, France; c Institut Européen des Membranes, Université de Montpellier II, 34000 Montpellier, France

## Abstract

The asymmetric unit of the title compound, C_17_H_25_NO_7_S, contains two independent mol­ecules, which are enanti­omers forming a hydrogen-bonded dimer associated with two *R*_2_^2^(7) patterns. In each mol­ecule, one ethyl group from the two available ethyl ester functional groups is disordered. In one mol­ecule, the ethyl group of the ester function from an α-carb­oxy­lic acid is positionally disordered over two sets of sites with occupancies of 0.66:0.34. In the second mol­ecule, it is the ethyl group in the γ-ester function that is disordered over two sets of sites with occupancies of 0.58:0.42.

## Related literature

For our studies on optically pure β-substituted β-hy­droxy aspartates as glutamate transporter blockers, see: Wehbe *et al.* (2003*a* ▶,*b* ▶,*c* ▶); Mekki *et al.* (2011*a* ▶,*b* ▶). For hydrogen-bond motifs, see: Etter (1990 ▶); Bernstein *et al.* (1995 ▶). For the visualization of non-covalent inter­actions, see: Johnson *et al.* (2010 ▶); *Jmol* (2011) ▶. For a description of the *Jmol* toolkit for the preparation of enhanced figures, see: McMahon & Hanson (2008 ▶).
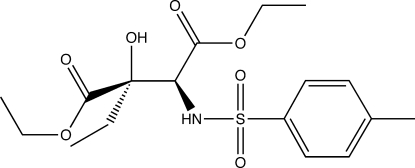


## Experimental

### 

#### Crystal data

C_17_H_25_NO_7_S
*M* *_r_* = 387.44
Triclinic, 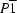

*a* = 9.5424 (3) Å
*b* = 12.2708 (4) Å
*c* = 18.2427 (5) Å
α = 90.800 (2)°
β = 91.153 (2)°
γ = 112.513 (3)°
*V* = 1972.36 (10) Å^3^
*Z* = 4
Cu *K*α radiation
μ = 1.79 mm^−1^
*T* = 173 K
0.27 × 0.24 × 0.12 mm

#### Data collection

Agilent Xcalibur Sapphire3 Gemini diffractometer
Absorption correction: multi-scan (*CrysAlis PRO*; Agilent, 2010 ▶) *T*_min_ = 0.110, *T*_max_ = 1.000
25194 measured reflections
6999 independent reflections
6214 reflections with *I* > 2σ(*I*)
*R*_int_ = 0.043

#### Refinement

*R*[*F* ^2^ > 2σ(*F* ^2^)] = 0.040
*wR*(*F* ^2^) = 0.114
*S* = 1.02
6999 reflections
527 parameters
81 restraints
H atoms treated by a mixture of independent and constrained refinement
Δρ_max_ = 0.34 e Å^−3^
Δρ_min_ = −0.31 e Å^−3^

### 

Data collection: *CrysAlis PRO* (Agilent, 2010 ▶); cell refinement: *CrysAlis PRO*; data reduction: *CrysAlis PRO*; program(s) used to solve structure: *SHELXS97* (Sheldrick, 2008 ▶); program(s) used to refine structure: *SHELXL97*; molecular graphics: *OLEX2* (Dolomanov *et al.*, 2009 ▶) and *Jmol* (Jmol, 2011 ▶); software used to prepare material for publication: *PLATON* (Spek, 2009 ▶) and *publCIF* (Westrip, 2010 ▶).

## Supplementary Material

Crystal structure: contains datablock(s) I, global. DOI: 10.1107/S1600536811029527/dn2708sup1.cif

Structure factors: contains datablock(s) I. DOI: 10.1107/S1600536811029527/dn2708Isup2.hkl

Supplementary material file. DOI: 10.1107/S1600536811029527/dn2708Isup3.cml

Additional supplementary materials:  crystallographic information; 3D view; checkCIF report

**Fig. 4.**
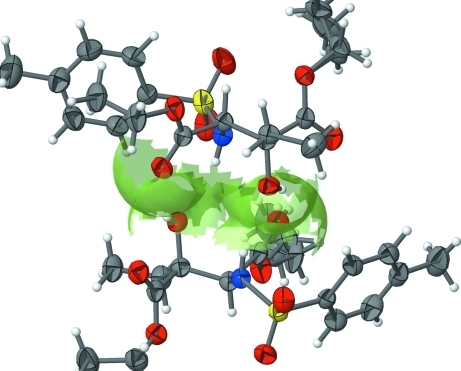
Enhanced Jmol view of the title compound showing displacement ellipsoids at the 50% probability level. Semi-translucent Van der Waals surfaces for donor H atoms and acceptors are displayed in green.

Enhanced figure: interactive version of Fig. 4

## Figures and Tables

**Table 1:** Hydrogen-bond geometry (Å, °)

*D*—H⋯*A*	*D*—H	H⋯*A*	*D*⋯*A*	*D*—H⋯*A*
N11—H11⋯O32	0.84 (2)	2.23 (2)	3.0426 (18)	162 (2)
O31—H31⋯O22	0.80 (2)	2.10 (2)	2.7747 (17)	142 (2)
N12—H12⋯O31	0.86 (2)	2.19 (2)	3.019 (2)	161 (2)
O32—H32⋯O21	0.81 (2)	2.15 (2)	2.8321 (17)	142 (2)

**Fig. 1.**
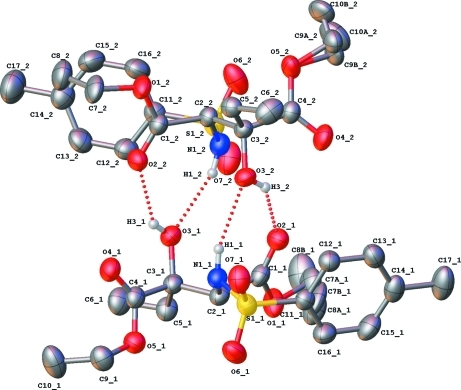
The asymmetric unit of the title compound with displacement ellipsoids for the non-hydrogen atoms drawn at the 50% probability level. Hydrogen bonds are indicated as dotted lines. Hydrogen atoms not involved in hydrogen bond interactions have been omitted for clarity.

**Fig. 2.**
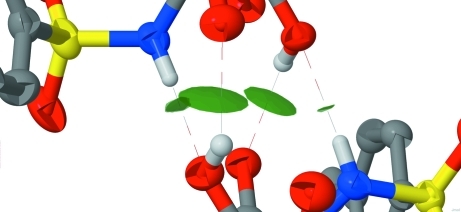
Van der Waals intersection surfaces (green) between nitrogen and oxygen hydrogen bond donors and oxygen acceptors of the two independent molecules in the asymmetric unit.

**Fig. 3.**
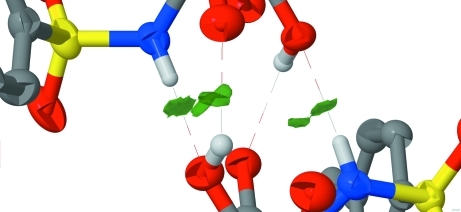
Non-covalent interaction surfaces (green) between nitrogen and oxygen hydrogen bond donors and oxygen acceptors of the two independent molecules in the asymmetric unit.

**Fig. 5.**
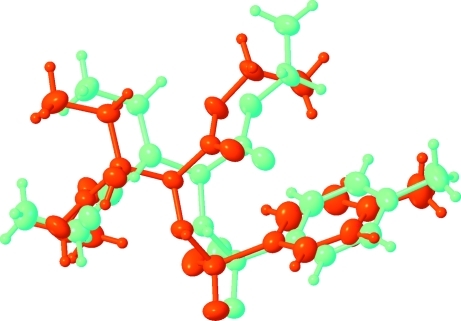
Best superposition of the two independent molecules in the asymmetric unit. Hydrogen bonds are omitted for clarity.

## supplementary crystallographic information

### Comment

In the present work, as a part of an on-going study of asymmetric syntheses of optically pure β*-*substituted β*-*hydroxy aspartates (Wehbe *et al.*, 2003*a*,*b*,*c*; Mekki *et al.*, 2011*a*,*b*), the structure of a new compound, *threo*-diethyl 2-ethyl-2-hydroxy-3-(4-methylphenylsulfonamido)succinate, is described. The key step of the synthesis is the regiospecific Sharpless aminohydroxylation on an ethyl fumarate derivative.

The crystal structure is made up by racemic dimers formed by two independent homochiral molecules ((2*S*,3*S*) and (2*R*,3*R*) for (I) and (II), respectively). They are bonded by non-covalent NH···O and OH···O hydrogen bonds (Fig. 1) forming two *R*^2^_2_(7) patterns (Etter, 1990; Bernstein *et al.*, 1995), where the H···O distances range from 2.10 (2) Å to 2.232 (19) Å and the *D*—H···O angles from 142 (2) to 161.9 (18) ° (Table 1). In order to get an idea of the relative strength of the NH···O and OH···O hydrogen bonds the intersection of the Van der Waals surfaces of donor hydrogen and acceptor was calculated using the program *Jmol* (*Jmol*, 2011; 'contact' command with 'full' and 'hbond' options). The resulting Fig. 2 shows clearly that the Van der Waals interaction zones between the hydroxyl groups and the carbonyl ester O atoms are more important than those between the hydroxyl groups and the secondary amine group. The latter interaction zones are much smaller than the former ones. A calculation based on the electron density and its derivatives (Johnson *et al.*, 2010; calculation done in *Jmol* using the 'contact' command with 'nci' and 'hbond' as options) gives slightly different results (Fig. 3), in the sense that one of the OH···O interactions appears to be negligible. The relevant Van der Waals surfaces may be inspected in the enhanced *Jmol* picture in Fig. 4. This pictorial view of the non-covalent interaction regions is not completely in agreement with what could be concluded from the directionality of the interaction which is greater for nitrogen as hydrogen bond donor than for oxygen (Table 1). The dimeric structure bears much similarity with those reported recently for the two concomitant β-benzyl β-hydroxy aspartate analogue polymorphs (Mekki *et al.*, 2011*a*).

The two independent homochiral molecules are very approximately related by a local inversion center between the two molecules. That this local center is only very approximate, can be clearly seen in Fig. 5, which shows the best superposition of the (2*S*,3*S*) molecule (I) and the (2*S*,3*S*) inversion center related molecule (II) as calculated with *Olex2* (Dolomanov *et al.*, 2009). The root-mean-squared deviation (considering the majority disordered parts only) between the two molecules is 0.780 Å. The main conformational differences between molecules (I) and (II) stem from the orientation of the ethyl ester moiety in both residues. This is well illustrated by the torsion angles C9—O5—C4—C3 (-4.2 (2)° and 173.6 (3)° for molecules (I) and (II), respectively) and C1–01-C7—C8 (-165.5 (3)° and -88.6 (2)° for molecules (I) and (II), respectively).

In both molecules, the S1—N1(H1)—C2 pseudo-torsion angle [140.2 (1)° for (I) and -143.3 (1)° for (II)] implies a slight pyramidalization of the sulfonamide moiety.

### Experimental

A solution of (0.04 mmole, 14.7 mg) K_2_[OsO_2_(OH)_4_] in water (2.5 ml) and chloramine-T (1.5 mmole, 423 mg) was added to a solution of diethyl 2-ethylfumarate (20 mg, 0.1 mmole) and (DHQD)_2_PHAL (0.05 mmole, 39 mg) in CH_3_CN (1.25 ml). After 1 h stirring at room temperature a second fraction of diethyl 2-ethylfumarate (180 mg, 0.9 mmole) in CH_3_CN (1.25 ml) was added to the reaction mixture. After 5 h, a solution of Na_2_SO_3_ (357 mg) in water (5.4 ml) was added an the reaction mixture was extracted 3 times with AcOEt (5.4 ml). The fraction was then washed with brine and dried under MgSO_4_. The solvent was removed and the title compound was recrystallized in cyclohexane by slow evaporation at ambient temperature yielding colourless crystals in the form of relatively large prisms.

### Refinement

All N-bound and O-bound H atoms were located in a difference Fourier maps and later restraint to a distance O–H = 0.82 (2) Å with *U*_iso_(H)=1.5*U*_eq_(O) and N–H = 0.88 (2) Å with *U*_iso_(H)=1.2*U_eq_*(N) in order to stabilize their coordinates during the final step of the refinement. All other H atoms were introduced at calculated positions and refined as riding atoms with C–H = 0.96–0.98 Å, with displacement parameters *U*_iso_(H) equal to 1.5*U*_eq_(C) for methyl and 1.2*U*_eq_(C) for all other H atoms. Restraints (SADI, SIMU, DELU) were used to stabilize the refinement of the disordered diethyl groups. The occupancies of the disordered parts were fixed during the final cycles of the refinements.

### Crystal data

**Table:**

C_17_H_25_NO_7_S	*Z* = 4
*M**_r_* = 387.44	*F*(000) = 824
Triclinic, *P*1	*D*_x_ = 1.305 Mg m^−^^3^
Hall symbol: -P 1	Cu *K*α radiation, λ = 1.54184 Å
*a* = 9.5424 (3) Å	Cell parameters from 6999 reflections
*b* = 12.2708 (4) Å	θ = 4.6–67.3°
*c* = 18.2427 (5) Å	µ = 1.79 mm^−^^1^
α = 90.800 (2)°	*T* = 173 K
β = 91.153 (2)°	Prism, colourless
γ = 112.513 (3)°	0.27 × 0.24 × 0.12 mm
*V* = 1972.36 (10) Å^3^	

### Data collection

**Table:**

Agilent Xcalibur Sapphire3 Gemini diffractometer	6999 independent reflections
Radiation source: Enhance (Cu) X-ray Source	6214 reflections with *I* > 2σ(*I*)
graphite	*R*_int_ = 0.043
Detector resolution: 16.0143 pixels mm^-1^	θ_max_ = 67.3°, θ_min_ = 4.6°
ω scans	*h* = −11→11
Absorption correction: multi-scan (*CrysAlis PRO*; Agilent, 2010)	*k* = −14→14
*T*_min_ = 0.110, *T*_max_ = 1.000	*l* = −21→21
25194 measured reflections	

### Refinement

**Table:**

Refinement on *F*^2^	Primary atom site location: structure-invariant direct methods
Least-squares matrix: full	Secondary atom site location: difference Fourier map
*R*[*F*^2^ > 2σ(*F*^2^)] = 0.040	Hydrogen site location: inferred from neighbouring sites
*wR*(*F*^2^) = 0.114	H atoms treated by a mixture of independent and constrained refinement
*S* = 1.02	*w* = 1/[σ^2^(*F*_o_^2^) + (0.066*P*)^2^ + 0.4378*P*] where *P* = (*F*_o_^2^ + 2*F*_c_^2^)/3
6999 reflections	(Δ/σ)_max_ = 0.001
527 parameters	Δρ_max_ = 0.34 e Å^−^^3^
81 restraints	Δρ_min_ = −0.31 e Å^−^^3^

### Special details

**Table:**

Experimental. *CrysAlis PRO* (Agilent, 2010); Empirical absorption correction using spherical harmonics, implemented in SCALE3 ABSPACK scaling algorithm.
Geometry. All e.s.d.'s (except the e.s.d. in the dihedral angle between two l.s. planes) are estimated using the full covariance matrix. The cell e.s.d.'s are taken into account individually in the estimation of e.s.d.'s in distances, angles and torsion angles; correlations between e.s.d.'s in cell parameters are only used when they are defined by crystal symmetry. An approximate (isotropic) treatment of cell e.s.d.'s is used for estimating e.s.d.'s involving l.s. planes.
Refinement. Refinement of *F*^2^ against ALL reflections. The weighted *R*-factor *wR* and goodness of fit *S* are based on *F*^2^, conventional *R*-factors *R* are based on *F*, with *F* set to zero for negative *F*^2^. The threshold expression of *F*^2^ > σ(*F*^2^) is used only for calculating *R*-factors(gt) *etc*. and is not relevant to the choice of reflections for refinement. *R*-factors based on *F*^2^ are statistically about twice as large as those based on *F*, and *R*- factors based on ALL data will be even larger.

### Fractional atomic coordinates and isotropic or equivalent isotropic displacement parameters (Å^2^)

**Table:**

	*x*	*y*	*z*	*U*_iso_*/*U*_eq_	Occ. (<1)
S11	0.49201 (5)	0.41250 (4)	0.16266 (2)	0.03996 (12)	
O11	0.36355 (15)	0.60190 (15)	0.33568 (7)	0.0597 (4)	
O21	0.48790 (14)	0.69378 (11)	0.23799 (7)	0.0461 (3)	
O31	0.77622 (13)	0.69982 (11)	0.32660 (7)	0.0436 (3)	
H31	0.852 (2)	0.702 (2)	0.3077 (13)	0.065*	
O41	0.90123 (14)	0.54137 (12)	0.31132 (7)	0.0494 (3)	
O51	0.69568 (14)	0.39398 (11)	0.35277 (7)	0.0455 (3)	
O61	0.43567 (17)	0.30752 (11)	0.20417 (8)	0.0552 (3)	
O71	0.57708 (15)	0.41810 (12)	0.09820 (7)	0.0516 (3)	
N11	0.60481 (15)	0.51686 (13)	0.21844 (7)	0.0377 (3)	
H11	0.656 (2)	0.5792 (15)	0.1966 (11)	0.045*	
C11	0.46431 (18)	0.61992 (16)	0.28350 (9)	0.0400 (4)	
C21	0.54737 (18)	0.53549 (15)	0.28902 (9)	0.0376 (3)	
H21	0.4746	0.4577	0.3062	0.045*	
C31	0.68397 (18)	0.58310 (15)	0.34482 (9)	0.0379 (3)	
C41	0.77443 (19)	0.50450 (16)	0.33431 (9)	0.0400 (4)	
C51	0.6352 (2)	0.58202 (18)	0.42443 (9)	0.0463 (4)	
H5A1	0.5827	0.6371	0.4301	0.056*	
H5B1	0.5622	0.5019	0.4355	0.056*	
C61	0.7683 (2)	0.6170 (2)	0.47904 (11)	0.0575 (5)	
H6A1	0.8154	0.5588	0.4767	0.086*	
H6B1	0.7321	0.6200	0.5286	0.086*	
H6C1	0.8431	0.6949	0.4670	0.086*	
C7A1	0.2915 (6)	0.6916 (5)	0.3353 (4)	0.0568 (13)	0.66
H7A1	0.3701	0.7717	0.3301	0.068*	0.66
H7B1	0.2179	0.6752	0.2935	0.068*	0.66
C8A1	0.2124 (4)	0.6844 (4)	0.40588 (19)	0.0621 (9)	0.66
H8A1	0.1381	0.6038	0.4117	0.093*	0.66
H8B1	0.1603	0.7394	0.4056	0.093*	0.66
H8C1	0.2869	0.7052	0.4467	0.093*	0.66
C7B1	0.2456 (11)	0.6449 (11)	0.3313 (8)	0.071 (3)	0.34
H7C1	0.1489	0.5876	0.3497	0.085*	0.34
H7D1	0.2295	0.6673	0.2808	0.085*	0.34
C8B1	0.3134 (11)	0.7484 (10)	0.3809 (5)	0.093 (3)	0.34
H8D1	0.3265	0.7224	0.4302	0.139*	0.34
H8E1	0.2465	0.7922	0.3827	0.139*	0.34
H8F1	0.4124	0.7996	0.3628	0.139*	0.34
C91	0.7677 (2)	0.30975 (18)	0.33901 (11)	0.0525 (4)	
H9A1	0.8184	0.3263	0.2913	0.063*	
H9B1	0.6889	0.2287	0.3362	0.063*	
C101	0.8821 (3)	0.3172 (2)	0.39851 (14)	0.0725 (7)	
H10A1	0.9676	0.3936	0.3967	0.109*	
H10B1	0.9188	0.2535	0.3913	0.109*	
H10C1	0.8347	0.3095	0.4463	0.109*	
C111	0.33817 (18)	0.45303 (14)	0.14075 (9)	0.0370 (3)	
C121	0.3501 (2)	0.52924 (16)	0.08434 (9)	0.0423 (4)	
H121	0.4370	0.5551	0.0547	0.051*	
C131	0.2333 (2)	0.56743 (17)	0.07165 (11)	0.0508 (4)	
H131	0.2408	0.6197	0.0327	0.061*	
C141	0.1055 (2)	0.53164 (17)	0.11419 (11)	0.0504 (4)	
C151	0.0970 (2)	0.4554 (2)	0.17044 (12)	0.0573 (5)	
H151	0.0105	0.4301	0.2004	0.069*	
C161	0.2117 (2)	0.41539 (18)	0.18398 (11)	0.0516 (4)	
H161	0.2039	0.3625	0.2226	0.062*	
C171	−0.0214 (3)	0.5743 (2)	0.10073 (16)	0.0741 (7)	
H17A1	−0.1189	0.5069	0.0997	0.111*	
H17B1	−0.0075	0.6141	0.0536	0.111*	
H17C1	−0.0201	0.6298	0.1402	0.111*	
S12	0.84903 (5)	1.02772 (4)	0.29860 (2)	0.04747 (13)	
O12	1.16142 (13)	0.94133 (13)	0.16081 (7)	0.0505 (3)	
O22	1.02729 (14)	0.80796 (11)	0.24124 (7)	0.0470 (3)	
O32	0.71903 (13)	0.74485 (10)	0.13370 (7)	0.0409 (3)	
H32	0.640 (2)	0.746 (2)	0.1483 (13)	0.061*	
O42	0.57282 (14)	0.88091 (13)	0.09183 (8)	0.0538 (3)	
O52	0.79035 (14)	1.03488 (11)	0.07151 (8)	0.0486 (3)	
O62	0.8723 (2)	1.12985 (13)	0.25634 (8)	0.0641 (4)	
O72	0.73105 (17)	0.98906 (16)	0.35091 (8)	0.0660 (4)	
N12	0.81369 (16)	0.91758 (14)	0.24099 (8)	0.0403 (3)	
H12	0.781 (2)	0.8512 (15)	0.2626 (11)	0.048*	
C12	1.03877 (18)	0.88414 (15)	0.19833 (9)	0.0376 (3)	
C22	0.91316 (17)	0.92874 (14)	0.17950 (9)	0.0353 (3)	
H22	0.9614	1.0140	0.1670	0.042*	
C32	0.81149 (17)	0.85996 (14)	0.11295 (9)	0.0340 (3)	
C42	0.70850 (18)	0.92560 (15)	0.09133 (9)	0.0381 (4)	
C52	0.90368 (19)	0.85057 (15)	0.04729 (9)	0.0389 (4)	
H5A2	0.9816	0.9296	0.0379	0.047*	
H5B2	0.9570	0.7979	0.0597	0.047*	
C62	0.8076 (2)	0.8036 (2)	−0.02228 (11)	0.0579 (5)	
H6A2	0.7248	0.7284	−0.0124	0.087*	
H6B2	0.8708	0.7914	−0.0607	0.087*	
H6C2	0.7651	0.8606	−0.0386	0.087*	
C72	1.2928 (2)	0.9086 (2)	0.17253 (11)	0.0564 (5)	
H7A2	1.3525	0.9224	0.1274	0.068*	
H7B2	1.2575	0.8236	0.1836	0.068*	
C82	1.3905 (2)	0.9804 (2)	0.23496 (14)	0.0684 (6)	
H8A2	1.4197	1.0645	0.2253	0.103*	
H8B2	1.4818	0.9626	0.2400	0.103*	
H8C2	1.3339	0.9612	0.2804	0.103*	
C9A2	0.7216 (7)	1.1129 (6)	0.0412 (3)	0.0498 (13)	0.58
H9A2	0.6213	1.0660	0.0182	0.060*	0.58
H9B2	0.7872	1.1633	0.0035	0.060*	0.58
C10A2	0.7047 (8)	1.1870 (5)	0.1027 (3)	0.0780 (14)	0.58
H10A2	0.6370	1.1364	0.1389	0.117*	0.58
H10B2	0.6617	1.2423	0.0839	0.117*	0.58
H10C2	0.8044	1.2313	0.1259	0.117*	0.58
C9B2	0.6878 (9)	1.1038 (7)	0.0679 (5)	0.0497 (18)	0.42
H9C2	0.6388	1.0954	0.0186	0.060*	0.42
H9D2	0.6080	1.0760	0.1049	0.060*	0.42
C10B2	0.7904 (7)	1.2279 (5)	0.0836 (4)	0.0624 (15)	0.42
H10D2	0.7326	1.2788	0.0802	0.094*	0.42
H10E2	0.8714	1.2524	0.0478	0.094*	0.42
H10F2	0.8351	1.2347	0.1331	0.094*	0.42
C112	1.0231 (2)	1.05241 (15)	0.34519 (9)	0.0417 (4)	
C122	1.0273 (2)	0.98059 (18)	0.40217 (10)	0.0511 (4)	
H122	0.9382	0.9163	0.4153	0.061*	
C132	1.1631 (3)	1.0037 (2)	0.43969 (11)	0.0589 (5)	
H132	1.1667	0.9545	0.4788	0.071*	
C142	1.2943 (2)	1.0971 (2)	0.42160 (11)	0.0571 (5)	
C152	1.2877 (3)	1.1647 (2)	0.36312 (13)	0.0642 (6)	
H152	1.3773	1.2276	0.3490	0.077*	
C162	1.1536 (3)	1.14287 (18)	0.32469 (12)	0.0576 (5)	
H162	1.1512	1.1900	0.2842	0.069*	
C172	1.4417 (3)	1.1235 (3)	0.46385 (17)	0.0910 (9)	
H17A2	1.4979	1.0804	0.4411	0.137*	
H17B2	1.4202	1.0988	0.5147	0.137*	
H17C2	1.5026	1.2084	0.4632	0.137*	

### Atomic displacement parameters (Å^2^)

**Table:**

	*U*^11^	*U*^22^	*U*^33^	*U*^12^	*U*^13^	*U*^23^
S11	0.0442 (2)	0.0407 (2)	0.0390 (2)	0.02082 (18)	−0.00107 (16)	−0.00119 (16)
O11	0.0464 (7)	0.1004 (11)	0.0453 (7)	0.0415 (8)	0.0135 (6)	0.0136 (7)
O21	0.0424 (7)	0.0480 (7)	0.0516 (7)	0.0211 (6)	0.0082 (5)	0.0059 (6)
O31	0.0354 (6)	0.0449 (7)	0.0483 (7)	0.0129 (5)	0.0031 (5)	0.0038 (5)
O41	0.0380 (7)	0.0594 (8)	0.0538 (7)	0.0218 (6)	0.0046 (5)	0.0051 (6)
O51	0.0443 (7)	0.0473 (7)	0.0473 (7)	0.0199 (6)	0.0049 (5)	0.0043 (5)
O61	0.0664 (9)	0.0404 (7)	0.0598 (8)	0.0218 (6)	−0.0082 (7)	0.0038 (6)
O71	0.0541 (7)	0.0655 (8)	0.0453 (7)	0.0347 (7)	−0.0003 (6)	−0.0098 (6)
N11	0.0351 (7)	0.0436 (8)	0.0351 (7)	0.0157 (6)	0.0040 (5)	0.0029 (6)
C11	0.0298 (8)	0.0521 (10)	0.0372 (8)	0.0147 (7)	0.0026 (6)	−0.0017 (7)
C21	0.0331 (8)	0.0440 (9)	0.0343 (8)	0.0130 (7)	0.0048 (6)	0.0041 (7)
C31	0.0338 (8)	0.0430 (9)	0.0363 (8)	0.0137 (7)	0.0034 (6)	0.0041 (7)
C41	0.0370 (9)	0.0505 (10)	0.0329 (8)	0.0173 (8)	0.0003 (6)	0.0023 (7)
C51	0.0443 (9)	0.0598 (11)	0.0371 (9)	0.0225 (9)	0.0038 (7)	−0.0004 (8)
C61	0.0570 (12)	0.0768 (14)	0.0392 (9)	0.0267 (11)	−0.0043 (8)	−0.0029 (9)
C7A1	0.043 (3)	0.081 (3)	0.059 (3)	0.038 (3)	0.011 (3)	0.003 (3)
C8A1	0.0473 (18)	0.090 (3)	0.0539 (18)	0.0325 (18)	0.0016 (14)	−0.0199 (17)
C7B1	0.027 (5)	0.127 (10)	0.060 (5)	0.031 (5)	0.002 (4)	0.000 (6)
C8B1	0.101 (7)	0.148 (8)	0.064 (5)	0.087 (7)	0.004 (5)	−0.023 (5)
C91	0.0594 (11)	0.0510 (11)	0.0532 (10)	0.0282 (9)	−0.0003 (9)	−0.0025 (8)
C101	0.0917 (17)	0.0723 (15)	0.0710 (14)	0.0523 (14)	−0.0224 (13)	−0.0108 (11)
C111	0.0365 (8)	0.0381 (8)	0.0355 (8)	0.0136 (7)	−0.0009 (6)	−0.0028 (6)
C121	0.0421 (9)	0.0459 (9)	0.0391 (8)	0.0168 (8)	0.0035 (7)	0.0038 (7)
C131	0.0544 (11)	0.0487 (10)	0.0521 (10)	0.0233 (9)	−0.0060 (8)	0.0019 (8)
C141	0.0436 (10)	0.0522 (11)	0.0578 (11)	0.0224 (9)	−0.0107 (8)	−0.0171 (9)
C151	0.0368 (9)	0.0736 (14)	0.0580 (11)	0.0172 (9)	0.0078 (8)	−0.0025 (10)
C161	0.0446 (10)	0.0577 (11)	0.0478 (10)	0.0135 (9)	0.0059 (8)	0.0118 (8)
C171	0.0594 (13)	0.0789 (16)	0.0958 (18)	0.0416 (12)	−0.0172 (12)	−0.0252 (13)
S12	0.0538 (3)	0.0588 (3)	0.0417 (2)	0.0352 (2)	−0.00150 (18)	−0.00783 (19)
O12	0.0307 (6)	0.0710 (9)	0.0533 (7)	0.0228 (6)	0.0078 (5)	0.0169 (6)
O22	0.0410 (7)	0.0504 (7)	0.0556 (7)	0.0234 (6)	0.0064 (5)	0.0122 (6)
O32	0.0347 (6)	0.0384 (6)	0.0473 (6)	0.0110 (5)	0.0080 (5)	0.0058 (5)
O42	0.0334 (7)	0.0666 (9)	0.0657 (8)	0.0239 (6)	0.0021 (6)	0.0016 (7)
O52	0.0450 (7)	0.0396 (7)	0.0665 (8)	0.0227 (6)	−0.0096 (6)	0.0006 (6)
O62	0.0951 (11)	0.0635 (9)	0.0549 (8)	0.0549 (9)	−0.0132 (7)	−0.0097 (7)
O72	0.0586 (8)	0.1001 (12)	0.0516 (8)	0.0444 (8)	0.0058 (6)	−0.0156 (8)
N12	0.0361 (7)	0.0474 (8)	0.0401 (7)	0.0190 (7)	0.0050 (6)	−0.0018 (6)
C12	0.0312 (8)	0.0421 (9)	0.0405 (8)	0.0152 (7)	0.0031 (6)	0.0007 (7)
C22	0.0305 (8)	0.0386 (8)	0.0390 (8)	0.0157 (7)	0.0041 (6)	0.0022 (6)
C32	0.0304 (7)	0.0340 (8)	0.0383 (8)	0.0130 (6)	0.0046 (6)	0.0025 (6)
C42	0.0351 (9)	0.0446 (9)	0.0375 (8)	0.0187 (7)	0.0005 (6)	−0.0044 (7)
C52	0.0395 (9)	0.0386 (8)	0.0422 (9)	0.0186 (7)	0.0076 (7)	0.0013 (7)
C62	0.0553 (11)	0.0633 (12)	0.0468 (10)	0.0137 (10)	0.0076 (9)	−0.0129 (9)
C72	0.0330 (9)	0.0871 (15)	0.0577 (11)	0.0321 (10)	0.0067 (8)	0.0061 (10)
C82	0.0431 (11)	0.0901 (17)	0.0765 (15)	0.0310 (11)	−0.0062 (10)	0.0021 (13)
C9A2	0.044 (3)	0.056 (3)	0.058 (3)	0.029 (2)	0.004 (2)	0.011 (2)
C10A2	0.114 (5)	0.071 (3)	0.076 (3)	0.064 (3)	0.013 (3)	0.010 (3)
C9B2	0.046 (4)	0.043 (3)	0.073 (5)	0.031 (3)	0.001 (3)	0.010 (4)
C10B2	0.069 (4)	0.041 (3)	0.088 (4)	0.032 (3)	0.008 (3)	0.013 (3)
C112	0.0488 (10)	0.0419 (9)	0.0374 (8)	0.0210 (8)	0.0004 (7)	−0.0026 (7)
C122	0.0528 (11)	0.0497 (10)	0.0469 (10)	0.0149 (9)	0.0033 (8)	0.0080 (8)
C132	0.0656 (13)	0.0682 (13)	0.0451 (10)	0.0278 (11)	−0.0011 (9)	0.0123 (9)
C142	0.0537 (11)	0.0657 (13)	0.0493 (10)	0.0203 (10)	−0.0027 (9)	−0.0041 (9)
C152	0.0546 (12)	0.0539 (12)	0.0713 (14)	0.0064 (10)	0.0014 (10)	0.0090 (10)
C162	0.0643 (13)	0.0478 (11)	0.0568 (11)	0.0168 (10)	0.0002 (9)	0.0140 (9)
C172	0.0648 (15)	0.119 (2)	0.0807 (18)	0.0267 (16)	−0.0178 (13)	0.0017 (16)

### Hydrogen-bond geometry (Å, °)

**Table:**

*D*—H···*A*	*D*—H	H···*A*	*D*···*A*	*D*—H···*A*
N11—H11···O32	0.84 (2)	2.23 (2)	3.0426 (18)	162.(2)
O31—H31···O22	0.80 (2)	2.10 (2)	2.7747 (17)	142 (2)
N12—H12···O31	0.86 (2)	2.19 (2)	3.019 (2)	161 (2)
O32—H32···O21	0.81 (2)	2.15 (2)	2.8321 (17)	142 (2)
